# Characterization of a conserved outer-membrane protein in non-typeable *Haemophilus influenzae* with an unidentified impact on phenotype

**DOI:** 10.1128/spectrum.02801-25

**Published:** 2026-03-16

**Authors:** Ashley J. Fraser, Oren Cooper, Brian M. Forde, Timothy F. Murphy, Michael P. Jennings, John M. Atack

**Affiliations:** 1 Institute for Biomedicine & Glycomics, Griffith University, Gold Coast, Queensland, Australia; 2 Institute for Molecular Bioscience, The University of Queensland 1974, Brisbane, Queensland, Australia; 3 Division of Infectious Diseases, Department of Medicine, University at Buffalo, State University of New York 12292 , Buffalo, New York, USA; University of Melbourne, Melbourne, Australia

**Keywords:** non-typeable haemophilus influenzae, vaccine, pathobiology

## Abstract

Non-typeable *Haemophilus influenzae* (NTHi) is an opportunistic bacterial pathogen of the human airway and is a cause of significant morbidity and mortality worldwide. No vaccine currently exists in part due to the incredible diversity and extreme variability exhibited both genetically and phenotypically between NTHi strains. For a vaccine to be successful, selection of appropriate antigens is key. Antigens need to show high conservation between strains, be present in most/all strains, and be stably expressed. The uncharacterized protein NTHI1101, initially identified from sequential isolates of NTHi from the sputum of COPD patients, demonstrated attributes of interest as a potential vaccine antigen: analysis of over 4,500 individual NTHi genomes demonstrated this protein was present in over 99% of strains, with over 80% amino acid identity. Further analysis predicted that NTHI1101 contained a lipoprotein signal sequence, meaning it was likely located in the outer membrane. An experimental determination of the location of the NTHI1101 protein demonstrated that although this protein was present in enriched outer-membrane fractions of NTHi cells, analysis with whole-cell enzyme-linked immunosorbent assays indicated it was not surface located. A variety of *in vitro* analyses with relevance to pathobiology demonstrated that the loss of the NTHI1101 protein had no impact on the phenotype of NTHi. Therefore, although this protein is highly conserved and almost universally present in NTHi, we conclude that the use of this protein as a target for novel therapeutics is perhaps not warranted.

**IMPORTANCE:**

Non-typeable *Haemophilus influenzae* (NTHi) is a major human pathogen for which there is no vaccine. Subunits for a rationally designed vaccine need to be conserved and present in almost all strains of an organism, be stably expressed, and be surface-located, so they will be “recognized” by the immune system. Our work sought to determine if a highly conserved NTHi protein, NTHI1101, was surface-located and required for pathobiology. Characterization showed that this protein was located in the outer membrane, but not present on the bacterial cell surface, and not required for key aspects of disease. We therefore conclude that NTHI1101 should not be further investigated as a vaccine candidate, so that ineffective antigens are not included in an NTHi vaccine.

## INTRODUCTION

Non-typeable *Haemophilus influenzae* (NTHi) is a human-adapted opportunistic pathogen that colonizes the human nasopharynx asymptomatically early in life (1). NTHi is an opportunistic pathogen, and one of the leading bacterial causes of global respiratory infections (2), including middle ear infection, or otitis media, in children, and chronic obstructive pulmonary disease (COPD) and community-acquired pneumonia in the elderly (3, 4). These infections can range from mild to severe and predominately affect vulnerable populations (5). Before the introduction of the *H. influenzae* serotype b (Hib) vaccine in 1993, NTHi rarely caused disease (6). However, the vaccine’s success generated a vacant ecological niche that NTHi rapidly occupied. Over the last 25 years, NTHi has become the most common cause of disease mediated by all *Haemophilus* species (1, 2, 5–7). NTHi is also increasingly responsible for severe invasive diseases such as meningitis, bacteremia, sepsis, and septicemia (7–9). These invasive NTHi infections have a case fatality ratio of up to 16%, with the highest number of cases seen in children under the age of 5 and adults aged 65 years or older (2, 6, 10, 11). Due to its substantial impact on health, the World Health Organization has classified NTHi as a priority pathogen (12).

Antibiotics are regularly used to treat NTHi infections; however, resistance is becoming increasingly prevalent. Globally, β-lactam-resistant NTHi strains are on the rise, with up to 55% of newly isolated strains resistant (11). Additionally, multi-drug-resistant (MDR) NTHi strains are emerging worldwide, exhibiting resistance to combinations of β-lactam, macrolides, quinolones, cefuroxime, levofloxacin, tetracycline, and trimethoprim-sulfamethoxazole (13). This increasing emergence of MDR NTHi is raising serious concern amongst health care professionals, highlighting the urgent need for an effective vaccine (3, 14, 15).

Rationally designed sub-unit vaccines have been widely discussed as the most viable method of vaccine design against NTHi (15–18). For the development of rationally designed subunit vaccines to be successful, the selection of appropriate antigens is critical. Potential antigens must be stably expressed, conserved, be found in all/most strains of the pathogen, be exposed to the host, and elicit a good immune response (17, 19). Many suitable targets are often selected from outer-membrane proteins (OMPs) that are key virulence factors. NTHi express multiple outer-membrane proteins that have been investigated as vaccine antigens (3, 14, 20, 21). However, many of these proteins exhibit high variability between strains, particularly in surface-exposed regions, and many strains lack the full repertoire of these proteins (16). Many NTHi outer-membrane proteins investigated as vaccine candidates are also subject to phase variation—a reversible, high-frequency on/off switching of gene expression (22). Therefore, this extensive genetic, antigenic, and phenotypic variability presents a major challenge for NTHi vaccine development, as selecting a variable protein as an antigen carries the risk of inducing an immune response that may not recognize this antigen in different NTHi strains or target a protein that may be entirely absent (not present in the genome or able to switch off expression). Therefore, identifying suitable antigens for NTHi is incredibly challenging. One approach for NTHi is reverse vaccinology: conserved stably expressed proteins can be identified bioinformatically, analyzed for conservation and presence, then screened *in vitro* for cellular localization and phenotypic role (15, 23). This approach was successfully used in developing the currently available Men4B vaccine against *Neisseria meningitidis* serogroup b (24, 25). Previous analysis of strains of NTHi isolated from patients suffering from COPD exacerbations identified a number of new potential antigenic targets from NTHi that exhibited sequence conservation in sequentially isolated strains over several months of *in vivo* carriage (26). One of these new antigenic targets was the protein NTHI1101 (NTHi strain 86-028NP locus designation). NTHI1101 is a predicted outer-membrane protein that shows high sequence conservation across all strains of NTHi, and the encoding gene contains no features associated with phase variation, making it a potentially ideal antigen. In this study, we aimed to determine the cellular location and characterize the phenotypic role of the NTHI1101 protein to assess its suitability as a component of a rationally designed subunit vaccine or a new therapeutic target for NTHi.

## RESULTS

### NTHI1101 is a highly conserved protein in NTHi

Initial analysis of 101 strains of NTHi from the sputum of COPD patients demonstrated that the NTHI1101 open reading frame (ORF) was present in all strains tested with a mean nucleotide sequence similarity of 98.7%, and did not significantly accumulate point mutations during prolonged carriage of these strains in the COPD lung (26). We subsequently extended this analysis to analyze 4,518 strains of NTHi with a publicly available genome sequence. This analysis demonstrated that the NTHI1101 protein was encoded in 99.7% of screened genomes (4,507/4,518), with an amino acid identity ≥80% and sequence coverage ≥80%.

### Generation of NTHI1101 knockout and complementation strains

**Fig 1:**
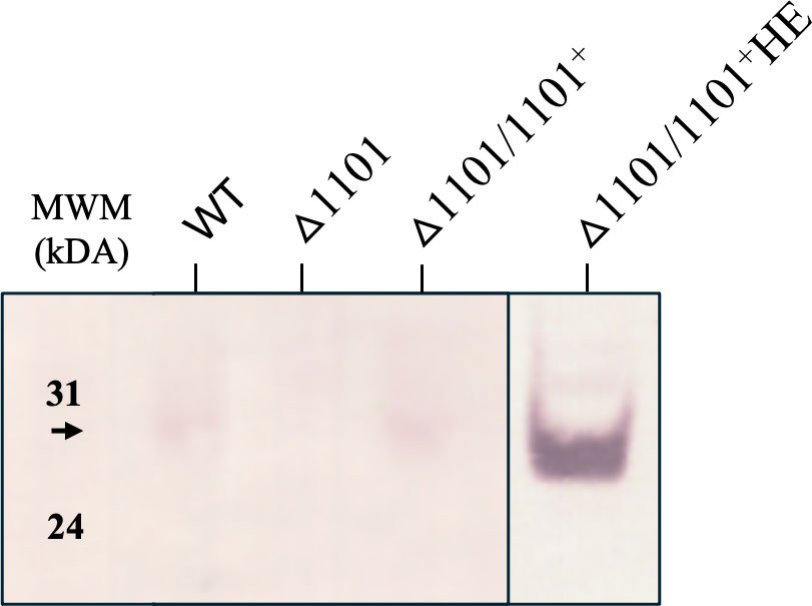
NTHI1101 knockout and complementation strains. Western blot analysis using whole-cell lysates of WT, △1101, △1101/1101^+^, and △1101/1101^+^HE strains. The Western blots are probed with anti-NTHI1101 antisera at a dilution of 1:500 and secondary antibody at a concentration of 1:10,000. The presence of NTHI1101 is detected in the WT and △1101/1101^+^ strain, as indicated by the black arrows, lack of expression is observed in the △1101 strain, and hyper-expression is seen in the △1101/1101^+^HE strain. Full Western blots are presented in Fig. S3.

A kanamycin resistance cassette was used to insertionally inactivate the *NTHI1101* gene in the NTHi wild-type (WT) strain 86-028NP, creating the △1101 strain. Absence of NTHI1101 expression was confirmed by Western blot (Fig. 1). To restore NTHI1101 function and generate a complementation strain, a functional copy of the *NTHI1101* gene plus flanking regions was reintroduced into the chromosome in *trans*. This construct also encoded functional flanking open reading frames (ORFs) *NTHI1103*, *NTHI1102*, and *NTHI1100*. According to the genome annotation, this entire four-gene region appeared to be an operon, which we confirmed with RT-PCR (Fig. S1). This complementation strain (△1101/1101^+^) demonstrated wild-type WT expression levels of NTHI1101 when analyzed by Western blot (Fig. 1). As the expression of NTHI1101 is very low, we generated a second complementation strain using just the *NTHI1101* ORF and ribosome binding site to increase the level of expression of this protein from the strong spectinomycin promoter present in the p601.1Sp NTHi complementation vector (27). This complementation strain (△1101/1101^+^HE; “hyper-expressing” complementation strain) showed very high expression of NTHI1101 compared to the WT and △1101/1101^+^ strain (Fig. 1) and would be useful in determining the location of this protein in enriched OMP fractions. Data from the analysis of this hyper-expressing complementation strain are presented in Fig. S5 through S8.

### NTHI1101 localizes with the outer membrane (OM)

**Fig 2:**
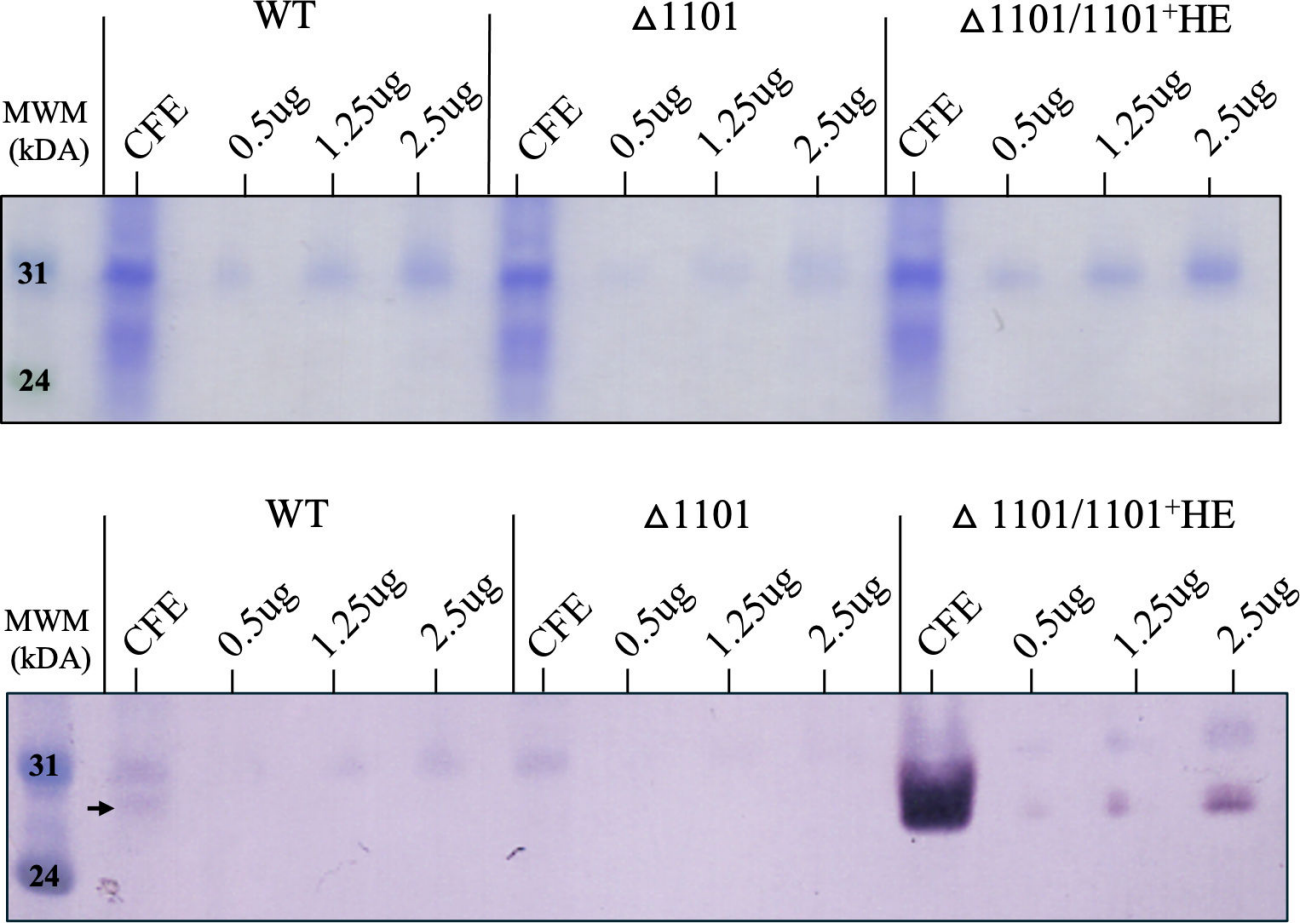
Enriched outer-membrane fractions. Coomassie (top) and Western blot (below) analysis using cell-free extracts (CFEs) and outer-membrane protein (OMP) enriched samples of WT, △1101, and △1101/1101^+^HE strains. The Western blot is probed with anti-NTHI1101 antisera at a dilution of 1:500 and secondary antibody at a concentration of 1:10,000. OMP fractions were loaded with increasing protein concentrations of 0.5, 1.25, and 2.5 µg. The presence of NTHI1101 is detected in the CFE of the WT, as indicated by the black arrow, but is not detectable in the WT OMP fractions. There is a complete lack of NTHI1101 in the △1101 strain. Hyper-expression of NTHI1101 is observed in the CFE of the △1101/1101^+^HE strain, and NTHI1101 is further detected in all OMP samples of the △1101/1101^+^HE strain. Full Western blots are presented in Fig. S4.

Bioinformatic analysis using SignalP version 6.0 (source https://services.healthtech.dtu.dk/services/SignalP-6.0/) predicted NTHI1101 to contain a lipoprotein signal sequence. To confirm that this prediction was correct, outer-membrane protein fractions were prepared from WT, △1101, and △1101/1101^+^HE strains using ultracentrifugation. Analysis using Western blot demonstrated the presence of NTHI1101 in the WT and △1101/1101^+^HE strains CFE, with no detectable presence in the △1101 strain CFE (Fig. 2). However, there was no evidence of the NTHI1101 protein in enriched outer-membrane protein fractions from WT cells, indicating a very low level of expression that we could not detect with Western blots. However, we could detect the NTHI1101 protein in enriched outer-membrane protein fractions from the △1101/1101^+^HE strain, providing good evidence that the NTHI1101 protein is associated with the outer membrane.

### NTHI1101 is not surface-exposed

**Fig 3:**
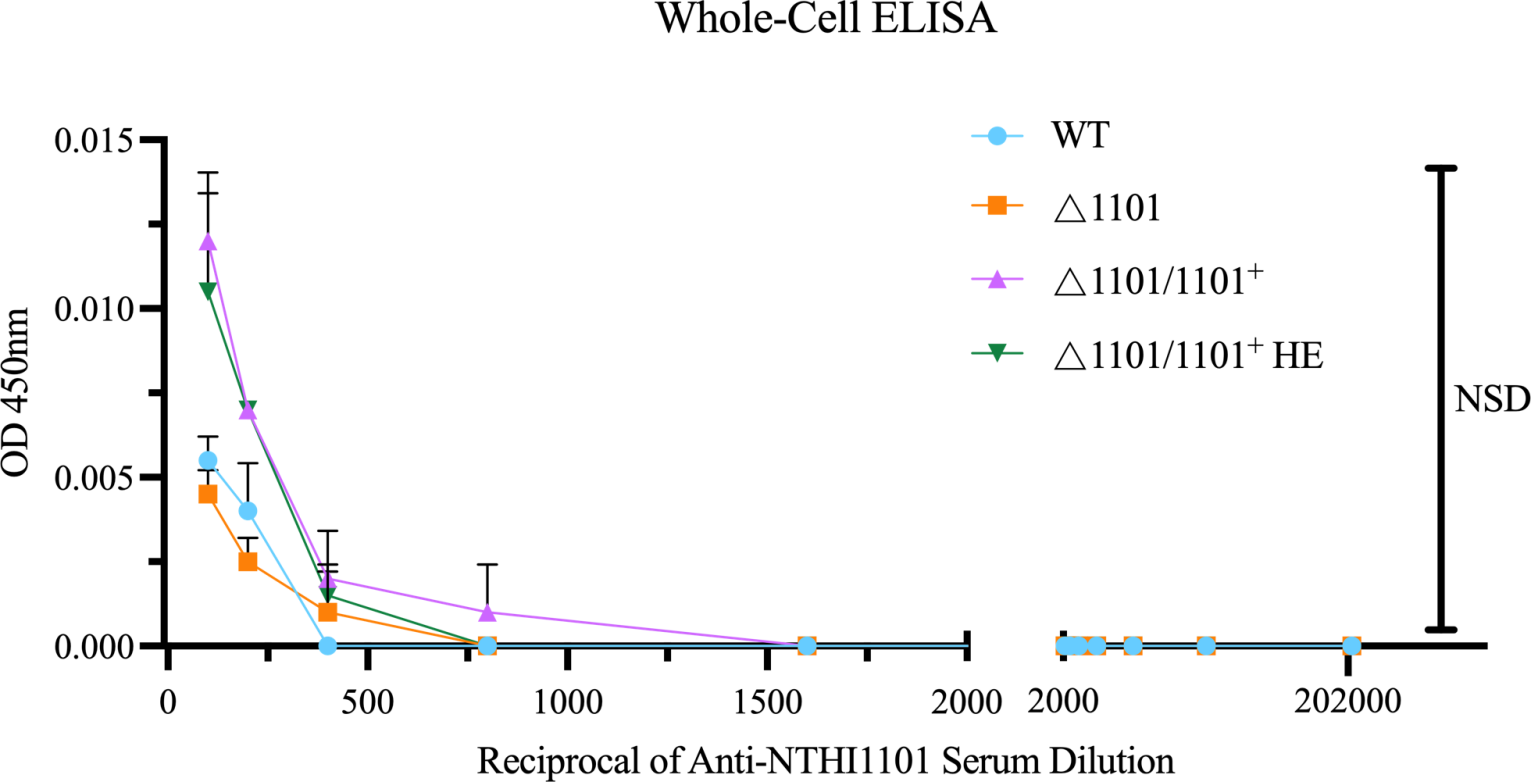
Whole-cell ELISA. Whole-cell ELISA of WT, △1101, △1101/1101^+^ and △1101/1101^+^HE strains, probed with primary antibody anti-NTHI1101 that was serially diluted twofold with a starting concentration of 1:100. Statistical analysis was performed using a one-way ANOVA. Error bars represent the standard deviation from mean values. NSD indicates no statistical difference between any of the strains. Enzyme-linked immunosorbent assay (ELISA).

To determine if the NTHI1101 protein is expressed on the bacterial surface, we conducted whole-cell enzyme-linked immunosorbent assays (ELISAs) using our knockout (KO) and complemented strains with anti-NTHI1101 anti-sera (Fig. 3). At all serum dilutions, absorbance levels were statistically the same as the knockout mutant, even with the △1101/1101^+^HE strain (Fig. 3), indicating NTHI1101 is not located on the surface of NTHi cells, and instead likely faces the periplasmic side of the OM, or is buried in the membrane and not exposed to the extracellular environment. Nevertheless, we hypothesized that this highly conserved protein would influence the properties of the OM, and would therefore affect the pathobiology of NTHi.

### NTHI1101 does not contribute to hyperosmotic stress resistance

**Fig 4:**
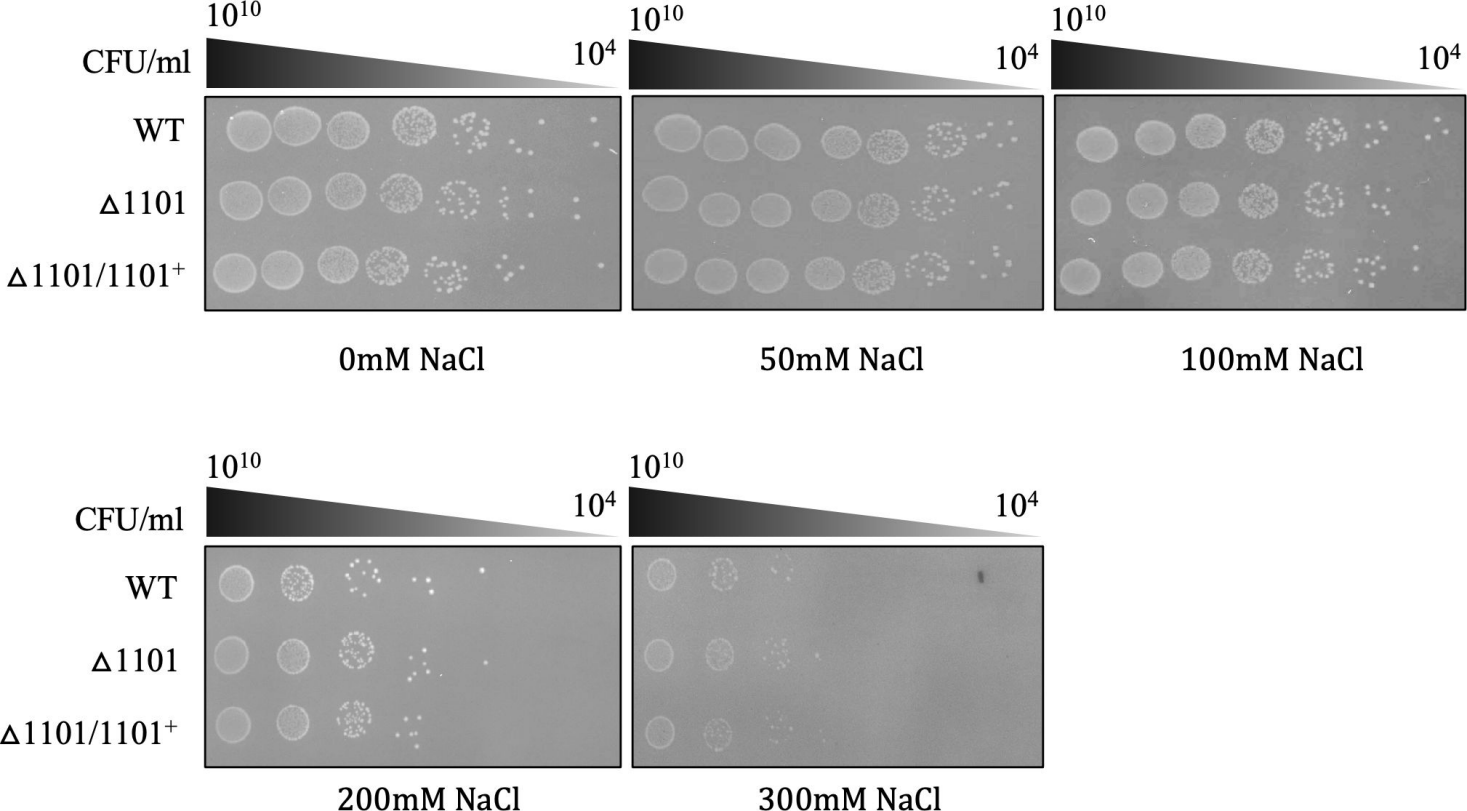
Hyperosmotic assay. Spot viability assay of WT, △1101, and △1101/1101^+^ strains to assess survival in response to hyperosmotic environment. Strains were serially diluted (10^10^–10^4^) and 2 µL dot plated onto sBHI agar (top left), sBHI agar supplemented with 50 mM NaCl (top middle) or 100 mM NaCl (top right) or 200 mM NaCl (bottom left) or 300 mM NaCl (bottom right). Images were captured with the BioRad ChemiDoc imaging system. Supplemented brain heart infusion (sBHI).

To assess whether the NTHI1101 protein is involved in membrane stability and integrity, we carried out hyperosmotic shock assays using supplemented brain heart infusion (sBHI) agar supplemented with NaCl at concentrations up to 300 mM. When grown on sBHI agar without additional NaCl, the WT, △1101, and △1101/1101^+^ strains demonstrated equal growth (Fig. 4). Similarly, on sBHI agar supplemented with 50–300 mM NaCl, there was no observed sensitivity or growth defect between the three strains, indicating NTHI1101 to be non-essential for hyperosmotic stress resistance (Fig. 4) and therefore does not play a role in stabilizing the outer membrane.

### NTHI1101 does not influence bacterial aggregation

**Fig 5:**
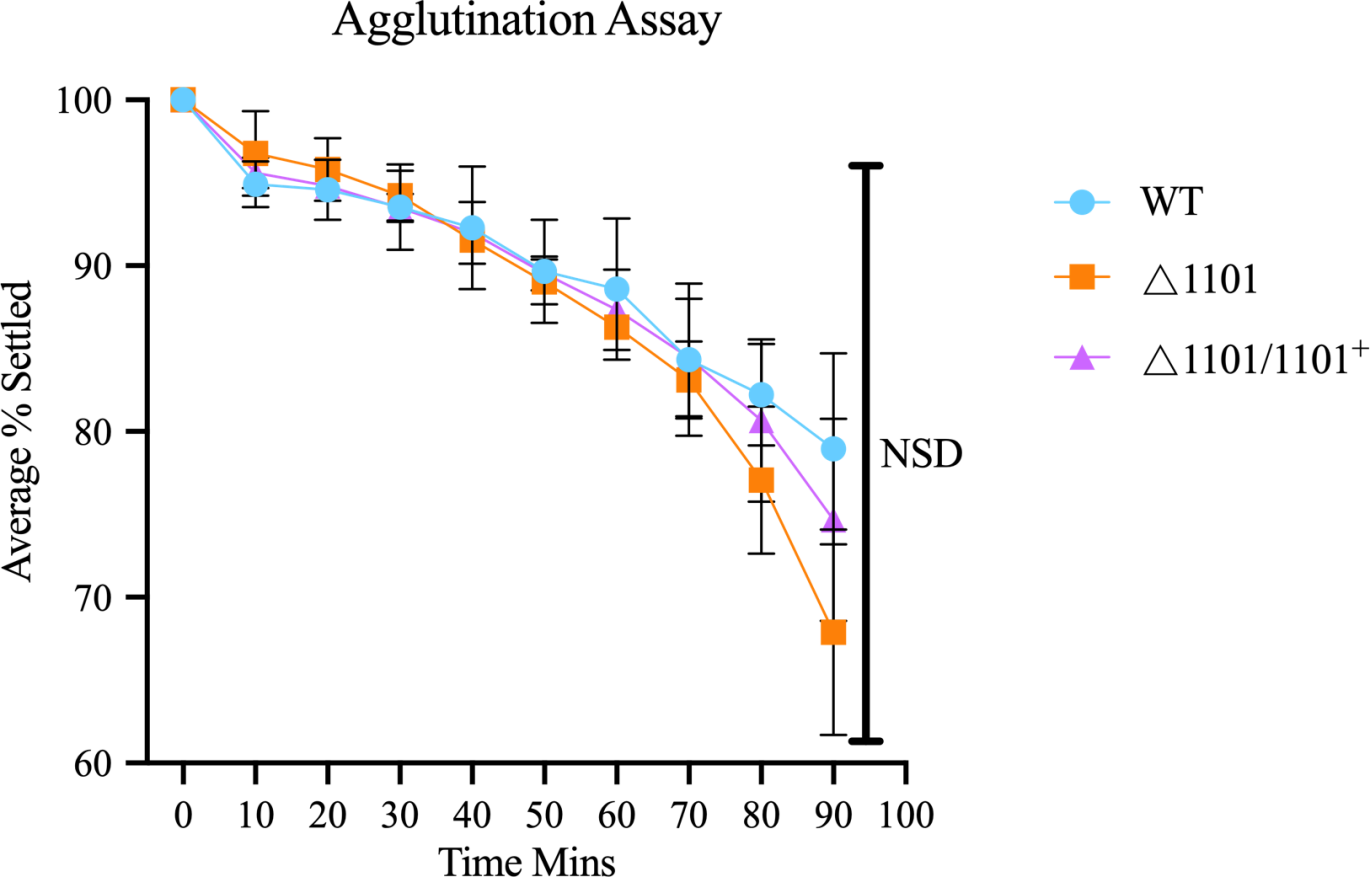
Settling assay. The rate of settling of WT, △1101, and △1101/1101^+^ strains was examined by monitoring the OD_600_ of static cultures over 90 min. Statistical analysis was performed using a one-way ANOVA. Error bars represent the standard deviation from mean values. NSD indicates no statistical difference between any of the strains.

To investigate the potential role of NTHI1101 in influencing cell-cell interactions, a settling assay was conducted. Strains WT, △1101, and △1101/1101^+^ were assessed for the rate at which bacterial cells settled over a 90-min time frame by measuring the absorbance at OD_600_. Results showed no difference in the settling rates of all three strains (Fig. 5).

### NTHI1101 does not have a role in biofilm formation

**Fig 6:**
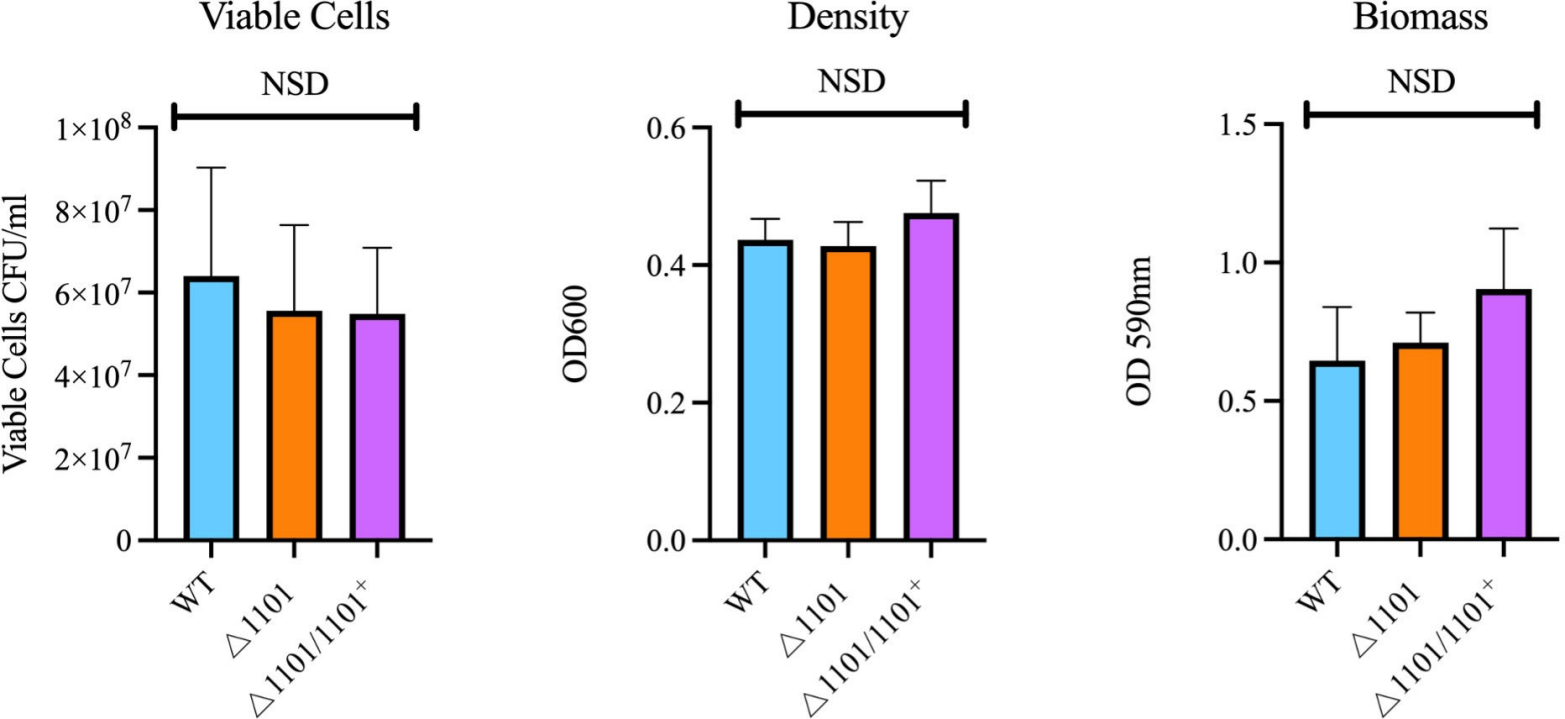
Biofilm assay. The biofilm formation capacity of WT, △1101, and △1101/1101^+^ was assessed through measures of viable cells (left), density (middle), and biomass (right) over a 48-h period. Statistical analysis was performed using an unpaired Student’s *t*-test, with *P* < 0.05 considered statistically significant. Error bars represent the standard deviation from mean values. NSD indicates no statistical difference between any of the strains.

To determine if NTHI1101 is involved in biofilm formation, biofilms from strains WT, △1101, and △1101/1101^+^ were grown for 48 h. Analysis of biomass (via OD_600_ and crystal violet staining) and cell viability demonstrated no significant difference between WT, △1101, and △1101/1101^+^ under these conditions (Fig. 6).

### NTHI1101 is not involved in the adherence to or invasion of A549 human lung cells

**Fig 7:**
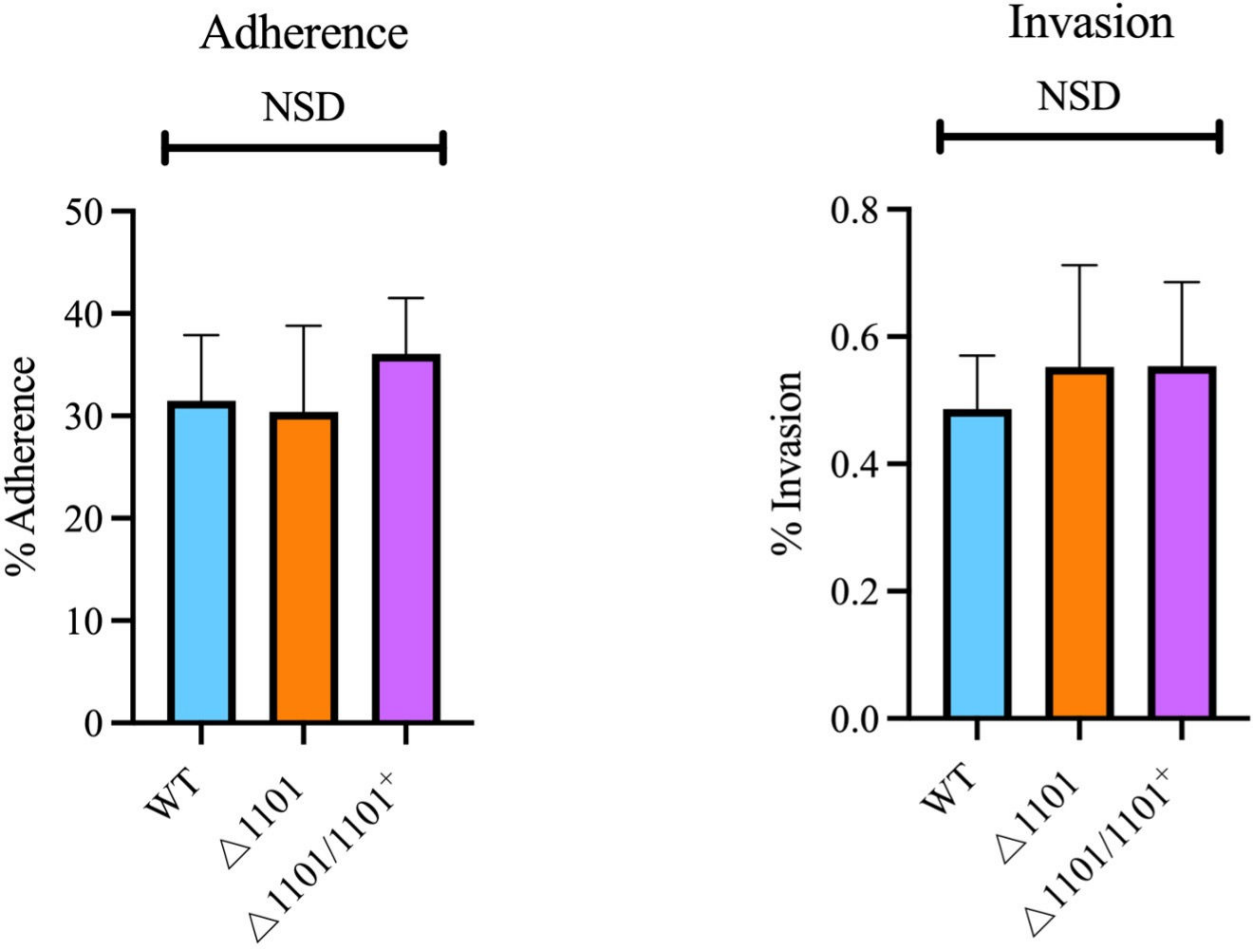
Adherence and invasion assay. The adherence and invasion capacity of WT, △1101, and △1101/1101^+^ was assessed using human A459 lung cells. Statistical analysis was performed using an unpaired Student’s *t*-test, with *P* < 0.05 considered statistically significant. Error bars represent the standard deviation from mean values. NSD indicates no statistical difference between any of the strains.

To assess if NTHI1101 is involved in adherence, the ability of the WT, △1101, and △1101/1101^+^ strains to adhere to and invade human A549 lung cells was analyzed. In the adherence assay, A549 cells were infected at a multiplicity of infection (MOI) of 25:1 (bacteria to lung cell) and incubated for 60 min. In parallel, invasion assays were conducted at the same MOI with a 120-min incubation period. Both assays showed no significant difference in the percentage of NTHi cells adhering to or invading A549 lung cells among the WT, △1101, and △1101/1101^+^ strains (Fig. 7).

## DISCUSSION

NTHi is a highly diverse pathogen, both phenotypically and genetically, making the identification of suitable antigens for vaccine development extremely challenging (3, 28). A suitable antigen must meet certain criteria to be considered for selection: candidates should ideally be stably expressed and conserved in all or most strains of the pathogen; candidates must be accessible to the host (such as being surface exposed); and ideally, candidates should have a role in colonization and/or disease (3, 17, 19).

NTHI1101, initially identified from sequential isolates of NTHi from the sputum of COPD patients (26, 29), demonstrated many of these attributes. The NTHI1101 protein was highly conserved, had a high antigenicity score, and was likely localized in the outer membrane (OM) (26). Analysis of 4,518 individual NTHi genomes demonstrated this protein was present in over 99% of strains, with over >80% amino acid identity. Further analysis predicted that NTHI1101 was a lipoprotein as the protein encoded a lipoprotein signal sequence (Fig. S2). We aimed to determine if NTHI1101 was surface-located, had a role in pathogenesis, and therefore demonstrate if further development of this conserved protein as a potential component of a subunit vaccine against NTHi was warranted.

Two complementation strains were generated for this study. The complemented strain, strain △1101/1101^+^, showed wild-type (WT) levels of NTHI1101 expression, and the second complementation strain, strain △1101/1101^+^HE, hyper-expressed the NTHI1101 protein (Fig. 1) and was useful in determining the location of the NTHI1101 protein. It is likely that the high expression levels in the △1101/1101^+^HE complementation strain were due to read-through from the upstream constitutively expressed spectinomycin resistance gene included in the NTHi complementation vector p601.1Sp. We demonstrated that the flanking ORFs *NTHI1102* and *NTHI1100* are in an operon with *NTHI1101* (Fig. S1) with the promoter upstream of *NTHI1102. NTHI1102* annotated as a hypothetical protein, and *NTHI1100* as a putative glutathionylspermidine synthase. It also appeared that *NTHI1103* (annotated as an enolase) was included in this operon (Fig. S1). Therefore, we included all four genes (NTHI1103–NTHI1100 inclusive) in the cloned region for generating our complemented strain. Including the promoter region upstream of the *NTHI1102* ORF and the *NTHI1103* ORF and the entire *NTHI1103–NTHI1100* region restored expression in the △1101/1101^+^ complementation strain to those comparable to WT expression levels of NTHI1101, but further work will be needed to validate this likely operonic nature.

We confirmed that the NTHI1101 protein colocalizes with the OM using enriched OM protein fractions. Although NTHI1101 was not detectable in the enriched OM proteins of the WT strain, the high expression of NTHI1101 in the Δ1101/1101^+^HE strain allowed for its detection in these enriched OM fractions. Using these same strains, whole-cell ELISAs demonstrated that the NTHI1101 protein was not located on the surface of the cell, even when the protein is present at much higher levels than seen in the WT (△1101/1101^+^HE). The likely periplasmic localization of NTHI1101 is a well-characterized phenomenon for gram-negative bacterial lipoproteins (30): for example, the majority of annotated lipoproteins in *Escherichia coli* associated with the OM are located in the periplasm (31); the lipoprotein Pal (peptidoglycan-associated lipoprotein) joins the gram-negative outer membrane to the peptidoglycan layer in the periplasm (32). Although we concluded that NTHI1101 protein is not on the surface of NTHi based on this data, we did hypothesize that this protein would have a role in the composition or stability of the outer membrane, and therefore would influence the phenotype of NTHi with respect to host-pathogen interactions, meaning the NTHI1101 protein may still be investigated as a new therapeutic target. Despite the use of multiple *in vitro* assays to determine the role of NTHI1101, we did not observe any effect on the phenotype of NTHi. Our adherence assays represent the total number of NTHi cells both adhered to A549 cells and intracellular NTHi, as we did not subtract the number of invaded cells from the total number of cells counted after our adherence assays. However, the very low number of intracellular NTHi (invaded NTHi) was so low, as this calculation made no difference to our conclusions, in that NTHI110 appears to have no role in interactions with human A549 cells. The lack of phenotype in adherence assays was also demonstrated with previous work using human bronchial epithelial NCI-H292 cells (26).

In conclusion, NTHi is a significant global pathogen and a major cause of morbidity and mortality worldwide (5), for which a vaccine is urgently needed. The selection of suitable antigens will be key to overcoming the immune-evasion mechanisms NTHi employs. Although the NTHI1101 protein was highly conserved, present in nearly all NTHi strains, stably expressed, and located in the OM, it was not located on the cell surface and had no discernible role in pathobiology. Further investigation into the role of NTHI1101 is required to determine what function this protein has in NTHi, but our work has demonstrated that it is likely not worth investigating further as a subunit vaccine candidate.

## MATERIALS AND METHODS

### Bacterial strains and growth conditions

NTHi strain 86-028NP was used as a model for this study. NTHi strain 86-028NP was originally isolated from the nasopharynx of a child with chronic otitis media (33). NTHi was grown in brain heart infusion (BHI) broth supplemented with 1% (vol/vol) hemin (4% triethanolamine, 1 mg/mL hemin, and 1 mg/mL l-Histidine) and 20 μg NAD^+^/mL (sBHI) and grown aerobically at 37°C with shaking at 200 rpm. For solid medium, 1.5% (wt/vol) agar was added to sBHI broth and grown at 37°C in 5% (vol/vol) CO_2_ atmosphere. sBHI medium was supplemented with 25 µg/mL kanamycin or 50 µg/mL spectinomycin, when required. *E. coli* used for cloning and protein expression was grown aerobically using Luria-Bertani (LB) at 37°C with shaking at 200 rpm. 1.5% (wt/vol) agar was added to LB broth for solid medium and grown at 37°C. LB medium was supplemented with 100 µg/mL ampicillin, 50 µg/mL kanamycin, or 50 µg/mL spectinomycin, when required.

### Analysis of NTHi presence and conservation

To investigate the prevalence and diversity of the NTHI1101 protein (NCBI accession number AAX87969.1 among *H. influenzae* strains, we screened a collection of publicly available *H. influenzae* genomes. A total of 5,785 *H. influenzae* genome assemblies were downloaded from GenBank (accessed on 1 June 2025). NTHi was identified *in silico* by screening the genome sequences of all assemblies against a database of capsule locus genes, as implemented in hicap v.1.0.4 using default parameters (34). A total of 4,518 genomes were identified not to carry a capsule locus and were classified as NTHi. The presence of NTHI1101 was determined using tBLASTn v.2.12.0+, using default parameters.

### Generation of NTHI1101 knockout mutant

To generate the NTHI1101 knockout (KO) mutant in NTHi strain wild-type (WT) 86-028NP, a kanamycin resistance cassette was used to insertionally inactive the *NTHI1101* gene. PCR using GoTaq DNA Polymerase was used to amplify a 1,498-bp fragment from WT NTHi 86-028NP genomic DNA containing the full *NTHI1101* gene including upstream and downstream flanking regions using primers NTHI1101-flank-F and NTHI1101-flank-R. This fragment was cloned into pGEM Teasy (Promega) to generate plasmid pGEM::NTHI1101-flank. Plasmids were then linearized with enzyme BsrG1 (New England BioLabs, NEB) that cuts 261 bp into the 576 bp NTHI1101 ORF, blunt ended using DNA Polymerase I, Large (Klenow) fragment (NEB) and dephosphorylated with Antarctic Phosphatase (NEB). Blunt-ended linear pGEM::NTHI1101-flank plasmids were then ligated with a blunt-ended kanamycin resistance cassette from pUC4kan using T4 DNA ligase (NEB) to generate pGEM::NTHI1101-flank::Kan^R^. Vector pGEM::NTHI1101-flank::Kan^R^ was linearized using enzyme AdhI (NEB) and transformed into NTHi strain WT 86-028NP using the M-IV method (35). Transformants were plated onto sBHI agar containing 20 µg/mL kanamycin, and colonies were confirmed via PCR using primers NTHI1101-outside-F and KanUPout-R. Confirmed colonies were grown overnight and checked for loss of expression via Western blot analysis using whole-cell lysates as described below. Strains were then designated NTHi 86-028NP::NTHI1101::KanR-KO, abbreviated to △1101.

### Generation of NTHI1101 hyper-expressing complementation strain

To generate the hyper-expressing complementation strain in this study, a functional *NTHI1101* gene was inserted back into the NTHi genome using the p601.1Sp complementation vector (27). PCR using KOD Hot Start Polymerase was used to amplify an 837-bp fragment from WT NTHi 86-028NP genomic DNA containing the full *NTHI1101* gene including a 255-bp upstream region that included the ribosome binding site and potential promoter region using primers NTHI1101-Comp-F and NTHI1101-OE-R. This fragment was ligated into the NTHi complementation vector p601.1Sp (27) to generate plasmid vector p601.1Sp::NTHI1101-comp_HE. Linear vectors were then transformed into strain NTHi 86-028NP::NTHI1101-Kan^R^ (△1101 strain generated above) using the M-IV method. Transformants were plated onto sBHI agar containing 50 µg/mL spectinomycin, and positive colonies were confirmed via PCR analysis using primers p601.1-SpecFOUT and NTHI1101-OE-R. Confirmed colonies were grown overnight and checked via Western blot analysis using whole-cell lysates as described below. Strains were designated NTHi 86-028NP::NTHI1101-compHE, abbreviated as △1101/1101^+^HE.

### Generation of NTHI1101 complementation strain

To generate a complementation strain with wild-type expression levels of NTHI1101, PCR using KOD Hot Start Polymerase was used to amplify a 4,078-bp fragment from WT NTHi 86-028NP genomic DNA containing the full *NTHI1101* gene including upstream and downstream regions which encoded ORFs *NTHI1103*, *NTHI1102*, and *NTHI1100*, using primers NTHI1101-XL-F and NTHI1101-XL-R. This region was inserted back into the genome *in trans* into the △1101 strain by cloning into the NTHi complementation vector p601.1Sp (27) as described above. Following transformation, spectinomycin-resistant colonies were screened via PCR using primers p601.1-SpecFOUT and NTHI1101-XL-R. Colonies were further checked via Western blot analysis using whole-cell lysates as described below. Strains were designated NTHi 86-028NP::NTHI1101-compXL, abbreviated as △1101/1101^+^.

### NTHI1101 protein overexpression, purification, and antisera production

To clone the *NTHI1101* gene for heterologous overexpression in *E. coli*, PCR using KOD Hot Start DNA polymerase (Merck-Millipore) and genomic DNA from NTHi strain WT 86-028NP was used with primers NTHI1101-OE-F and NTHI1101-OE-R to generate a 500-bp product. PCR products were then digested with NdeI and BamHI (NEB) and ligated into the pET15b vector digested with the same enzymes to clone the gene in frame with the N-terminal 6× polyHistidine tag. Following transformation into DH5alpha *E. coli* colonies were screened with PCR using GoTaq DNA Polymerase (Promega), using primers T7F and NTHI1101-OE-R. Vectors were verified by sequencing using BigDye 3.1, with Sanger Sequencing performed by the Griffith University DNA Sequencing Facility (GUDSF). This plasmid was designated pET15b::NTHI1101-OE. Plasmids were miniprepped (Promega Wizard miniprep kit) and transformed into chemically competent BL21 *E. coli* cells via heat shock. Overexpression was carried out with autoinduction using 1 L of ZYM-5052 media (36) overnight at 37°C with 200 rpm shaking. Cells were pelleted and resuspended in 1× binding buffer (50 mM NaPO_4_ and 300 mM NaCl [pH 7.4]), treated with DNase and lysozyme (10 µg/mL) then stored overnight at −20°C. To purify the NTHI1101 protein, pellets were thawed and lysed via sonication until transparent (30 s ON, 1 min OFF × 15 rounds at 30% power using the ultrasonic processor Sonics & Materials VCX500-220 V, Mereck). 0.1% (wt/vol) sodium dodecyl sulfate (SDS) was added following sonication, gently mixed at room temperature (RT) for 15 min, and cell debris pelleted at 6,000 × *g* for 5 min. Purification was carried out using gravity flow metal affinity resin (TALON resin, Takara) in 1× binding buffer using the soluble fraction. NTHI1101 protein was eluted from the TALON resin using stepwise concentrations of imidazole in 1× binding buffer. Following purification, fractions were analyzed by SDS-PAGE and Coomassie staining. Fractions containing pure NTHI1101 protein were pooled and concentrated using centrifugal concentrations (3 kDa cutoff; EMD Millipore) at 4,000 × *g* at 4°C. Concentrated protein was buffer exchanged into 1× PBS using the same centrifugal concentrator. Pure NTHI1101 protein was quantified using an extinction coefficient of 10.095 mM^−1^ cm^−1^ and molecular weight of 20.126 kDa (based on the sequence of the NTHI1101 protein + 6× polyHistidine tag). Pure protein was sent to the Walter and Eliza Hall (WEHI) Institute of Medical Research, Melbourne, Australia for the generation of anti-NTHI1101 anti-sera using rabbits.

### Western blotting and Coomassie staining

NTHi cultures were normalized to OD_600_ 4.0 in 1× phosphate-buffered saline (PBS), an appropriate volume of 4× LDS sample buffer (Novex) containing 10% (vol/vol) β-mercaptoethanol was added, and samples heated at 90°C for 30 min to lyse cells and denature proteins. Samples were run on 4–12% Bis-Tris pre-cast gels (Novex) using MES buffer (Sigma-Aldrich) at 150 V for 45 min. Gels for Coomassie staining were incubated in Coomassie blue-dye stain (40% [vol/vol] methanol, 10% [vol/vol] acetic acid, and 0.1% [wt/vol] Coomassie brilliant blue R dye) with shaking and destained in a solution of 40% (vol/vol) methanol and 10% (vol/vol) acetic acid to visualize protein bands. For Western blotting, protein was transferred to nitrocellulose membrane (Bio-Rad) at 15 V for 70 min and blocked with 5% (wt/vol) skim milk in 1× Tris-buffered saline with Tween 20 (TBST) with shaking. All blots were probed with anti-NTHI1101 antisera at a dilution of 1:500, and secondary antibody (anti-rabbit IgG [whole molecule] – alkaline phosphatase, Sigma-Aldrich) at a concentration of 1:10,000, unless otherwise stated.

### Preparation of outer-membrane proteins (OMPs)

Cultures of NTHi were grown in sBHI to mid log (OD_600_ 0.5) and harvested at 4,500 × *g* for 15 min at 4°C, freeze-thawed, then resuspended in 4 mL 10 mM Tris pH 8.0 and lysed further using sonication until transparent (as described above). Debris was pelleted as above and supernatant re-centrifuged. Sarcosyl (0.1%) (wt/vol) was added and incubated at 25°C for 30 min. Supernatants were then centrifuged at 35,000 × *g* for 90 min at 4°C. Pellets were resuspended in 4 mL 10 mM Tris pH 8.0 with Sarcosyl (0.1%) (wt/vol) and incubation and ultra-centrifugation steps repeated as above. Final pellets containing the OMP-enriched fractions were resuspended in 100 µL Tris pH 8.0, and the protein concentration quantified using a BCA protein assay kit (Thermo Scientific). OMP preparations were analyzed via SDS-PAGE and Western blot (as described above).

### Whole-cell enzyme-linked immunosorbent assays (ELISAs)

ELISAs were performed in 96-well Maxisorb plates (NUNC; Thermo Scientific). NTHi strains were grown to mid-log (OD_600_ = 0.5), washed, and resuspended in 1× PBS to an OD_600_ of 0.2. Wells were coated with 100 µL/well per strain in triplicate and dried overnight in a laminar flow hood. Plates were blocked with 150 µL/well of 3% (wt/vol) bovine serum albumin (BSA, Thermo Scientific) in 1× PBS for 1.5 h at RT, then washed four times with 1× PBS-Tween20 (PBS-T). Primary antibody anti-NTHI1101 was serially diluted twofold using 1× PBS with a starting concentration of 1:100, then added at 100 µL/well and incubated for 1.5 h at RT. Plates were then washed four times with 1× PBS-T. Secondary antibody (goat anti-rabbit horseradish peroxidase conjugate, Sigma-Aldrich) was used at a concentration of 1:10,000 in PBS and added at 100 µL/well and incubated for 1 h at RT. Plates were then washed four times with 1× PBS-T. 50 µL/well of TMB Single Substrate Solution (3,3′,5,5′-Tetramethylbenzidine, Invitrogen) was added and incubated for 30 min at RT. Reactions were stopped using 50 µL of 0.25 N HCl, and absorbance of each well was measured at 450 nm using a plate reader (TECAN Infinite 200 PRO).

### Hyperosmotic assay

NTHi strains were grown to mid-log (OD_600_ = 0.5), washed, and resuspended in 1× PBS to an OD_600_ of 1.0 and 10-fold serially diluted. In triplicate, 2 µL from each dilution was dot plated onto sBHI agar, and sBHI agar supplemented with between 50 and 300 mM NaCl. Plates were incubated overnight at 37°C with 5% (vol/vol) CO_2_.

### Settling assay

NTHi strains were grown to mid-log (OD_600_ = 0.5), washed, and resuspended in 1× PBS to an OD_600_ of 1.0. Triplicate 2 mL samples of each NTHi strain were aliquoted into cuvettes and monitored for 90 min, with absorbance readings taken at time (*T*), T0, T5, T10, T15, T20, T30, T45, T60, T70, T80, and T90 min using a spectrophotometer at a wavelength 600 nm.

### Biofilm assay

Biofilm assays were conducted and assessed using OD_600_, colony-forming units/mL (CFU/mL), and crystal violet analysis, all run in parallel with NTHi strains using 24-well flat-bottom tissue culture plates (Thermo Scientific) in triplicate per strain. Strains were grown to mid-log (OD_600_ = 0.5) and diluted to achieve a final inoculum of 2.5 × 10^5^ CFU per well. Plates were then incubated at 37°C with 5% (vol/vol) CO_2_ for a total of 48 h with a media change after the first 24 h. For the OD_600_ and CFU/mL wells: media were removed, and biofilms resuspended in 1 mL sBHI, with a 100 µL sample serially diluted and dot plated onto sBHI agar, grown overnight and used to calculate CFU/mL. The remaining sample was used to record the absorbance on a spectrophotometer at a wavelength of 600 nm. For the crystal violet wells, media were removed and the wells were washed with 1 mL of Milli-Q water three times before a 15-min incubation with 1 mL of crystal violet solution ([10%] [vol/vol] methanol and [1%] [vol/vol] crystal violet [Sigma-Aldrich]). Following this, the wells were washed as above, dried in a laminar flow hood, then incubated with 1 mL ethanol (95%) for 15 min, then scanned at 590 nm to assess biomass.

### Adherence assay

Adherence of NTHi strains to A549 human respiratory alveolar epithelial cells was carried out as follows: A549 cells were cultured to confluency in T-75 flasks in 1× RPMI 1640 media (Thermo Fisher Scientific) supplemented with 10% (vol/vol) fetal bovine serum (Thermo Fisher Scientific; supplemented RPMI, sRPMI) at 37 °C in a humidified atmosphere containing 5% CO_2_. Once cultures reached approximately 80% confluence, cells were washed with 1× PBS and detached using 1× TrypLE Express Enzyme (Gibco). Cell density was determined using a Neubauer hemocytometer, and cells were seeded into 96-well flat-bottom plates at a density of 2 × 10⁴ cells per well in sRPMI, then grown to 100% confluency, and washed in RPMI prior to adding bacteria. NTHi strains were cultured to mid-log phase (OD_600_ = 0.5), harvested by centrifugation at 3,000 × *g* for 10 min at room temperature, washed once in sRPMI, and resuspended in sRPMI to achieve a multiplicity of infection (MOI) of 25:1. Bacteria were added to the A549 cell monolayers in quadruplicate. Co-cultures were incubated for 1 h at 37 °C with 5% CO_2_. After incubation, cells were washed three times with supplemented brain heart infusion (sBHI) broth to remove non-adherent bacteria. Adherent bacteria were released by treating cells with TrypLE (Thermo Fisher Scientific) and vigorously pipetting to both lyse the A549 cells and ensure an even bacterial suspension, and then serially diluted 1:2 in sBHI using polypropylene 96-well plates, with vigorous pipetting during all steps. Aliquots (10 µL) of each dilution were spotted in triplicate onto sBHI agar plates and incubated for 36 h at 37 °C with 5% CO_2_. Colonies were counted manually using a light box. Data were analyzed using Microsoft Excel and GraphPad Prism (v10). The percentage adherence was calculated by comparing the number of colony-forming units (CFU) recovered to the number of CFU in the initial inoculum.

### Invasion assay

Invasion assays were performed in parallel with adherence assays, with the following modifications: after a 2-h incubation with NTHi at 37°C and 5% CO_2_, cells were treated with gentamicin (100 µg/mL) for 1 h to kill extracellular bacteria. Monolayers were then washed three times with sBHI to remove non-invaded and dead bacteria. Cells were subsequently dissociated with TrypLE, serially diluted in BHI, and plated as described above. The percentage of invasion was calculated as the CFU recovered relative to the CFU in the initial inoculum.

## SUPPLEMENTAL MATERIAL

Supplemental figures and table spectrum.02801-25-s0001.pdf Figures S1 to S8 and Table S1.

ASM does not own the copyrights to Supplemental Material that may be linked to, or accessed through, an article. The authors have granted ASM a non-exclusive, world-wide license to publish the Supplemental Material files. Please contact the corresponding author directly for reuse.

## References

[1] Chatziparasidis G, Kantar A, Grimwood K. 2023. Pathogenesis of nontypeable Haemophilus influenzae infections in chronic suppurative lung disease. Pediatr Pulmonol 58:1849–1860. doi:10.1002/ppul.26446 37133207

[2] Cerquetti M, Giufrè M. 2016. Why we need a vaccine for non-typeable Haemophilus influenzae. Hum Vaccin Immunother 12:2357–2361. doi:10.1080/21645515.2016.1174354 27171854 PMC5027700

[3] Behrouzi A, Vaziri F, Rahimi-Jamnani F, Afrough P, Rahbar M, Satarian F, Siadat SD. 2017. Vaccine candidates against nontypeable Haemophilus influenzae: a review. Iran Biomed J 21:69–76. doi:10.18869/acadpub.ibj.21.2.69 28088130 PMC5274713

[4] Briel M, Spoorenberg SMC, Snijders D, Torres A, Fernandez-Serrano S, Meduri GU, Gabarrús A, Blum CA, Confalonieri M, Kasenda B, Siemieniuk RAC, Boersma W, Bos WJW, Christ-Crain M, Ovidius Study Group, Capisce Study Group, STEP Study Group. 2018. Corticosteroids in patients hospitalized with community-acquired pneumonia: systematic review and individual patient data metaanalysis. Clin Infect Dis 66:346–354. doi:10.1093/cid/cix801 29020323

[5] Mahammed A, Damtie D, Sema T, Abdilahi Z. 2023. A global review of invasive haemophilus influenzae disease from 2000-2023: current status, challenges and future perspectives. Research Square. doi:10.21203/rs.3.rs-3412671/v1

[6] Agrawal A, Murphy TF. 2011. Haemophilus influenzae infections in the H. influenzae type b conjugate vaccine era. J Clin Microbiol 49:3728–3732. doi:10.1128/JCM.05476-11 21900515 PMC3209133

[7] Langereis JD, de Jonge MI. 2015. Invasive disease caused by nontypeable Haemophilus influenzae. Emerg Infect Dis 21:1711–1718. doi:10.3201/eid2110.150004 26407156 PMC4593434

[8] Ito T, Shibata H, Nakazawa M, Myokai M, Ikegaya K, Tsuchiya K, Kamimaki T. 2011. Meningitis and septicemia caused by nontypeable Haemophilus influenzae in a previously healthy 2-year-old girl. J Infect Chemother 17:559–562. doi:10.1007/s10156-011-0213-6 21286774 PMC3156912

[9] Slack MPE. 2017. The evidence for non-typeable Haemophilus influenzae as a causative agent of childhood pneumonia. Pneumonia (Nathan) 9:9. doi:10.1186/s41479-017-0033-2 28702311 PMC5483294

[10] King PT, Sharma R. 2015. The lung immune response to nontypeable haemophilus influenzae (lung immunity to NTHi). J Immunol Res 2015:706376. doi:10.1155/2015/706376 26114124 PMC4465770

[11] Van Eldere J, Slack MPE, Ladhani S, Cripps AW. 2014. Non-typeable Haemophilus influenzae, an under-recognised pathogen. Lancet Infect Dis 14:1281–1292. doi:10.1016/S1473-3099(14)70734-0 25012226

[12] WHO (2024). 2024. WHO bacterial priority pathogens list, 2024: bacterial pathogens of public health importance to guide research, development and strategies to prevent and control antimicrobial resistance. World Health Organisation.

[13] Su P-Y, Cheng W-H, Ho C-H. 2023. Molecular characterization of multidrug-resistant non-typeable Haemophilus influenzae with high-level resistance to cefuroxime, levofloxacin, and trimethoprim-sulfamethoxazole. BMC Microbiol 23:178. doi:10.1186/s12866-023-02926-6 37407940 PMC10320927

[14] Su Y-C, Kadari M, Straw ML, Janoušková M, Jonsson S, Thofte O, Jalalvand F, Matuschek E, Sandblad L, Végvári Á, Zubarev RA, Riesbeck K. 2023. Non-typeable Haemophilus influenzae major outer membrane protein P5 contributes to bacterial membrane stability, and affects the membrane protein composition crucial for interactions with the human host. Front Cell Infect Microbiol 13:1085908. doi:10.3389/fcimb.2023.1085908 37305414 PMC10250671

[15] Greenwood B. 2014. The contribution of vaccination to global health: past, present and future. Philos Trans R Soc Lond B Biol Sci 369:20130433. doi:10.1098/rstb.2013.0433 24821919 PMC4024226

[16] Jalalvand F, Riesbeck K. 2018. Update on non-typeable Haemophilus influenzae-mediated disease and vaccine development. Expert Rev Vaccines 17:503–512. doi:10.1080/14760584.2018.1484286 29863956

[17] Pettigrew MM, Alderson MR, Bakaletz LO, Barenkamp SJ, Hakansson AP, Mason KM, Nokso-Koivisto J, Patel J, Pelton SI, Murphy TF. 2017. Panel 6: vaccines. Otolaryngol Head Neck Surg 156:S76–S87. doi:10.1177/0194599816632178 28372533 PMC5505493

[18] Tan A, Atack JM, Jennings MP, Seib KL. 2016. The capricious nature of bacterial pathogens: phasevarions and vaccine development. Front Immunol 7:586. doi:10.3389/fimmu.2016.00586 28018352 PMC5149525

[19] Cunningham AL, Garçon N, Leo O, Friedland LR, Strugnell R, Laupèze B, Doherty M, Stern P. 2016. Vaccine development: from concept to early clinical testing. Vaccine (Auckl) 34:6655–6664. doi:10.1016/j.vaccine.2016.10.016 27769596

[20] Duell BL, Su Y-C, Riesbeck K. 2016. Host-pathogen interactions of nontypeable Haemophilus influenzae: from commensal to pathogen. FEBS Lett 590:3840–3853. doi:10.1002/1873-3468.12351 27508518

[21] Osman KL, Jefferies JM, Woelk CH, Cleary DW, Clarke SC. 2018. The adhesins of non-typeable Haemophilus influenzae. Expert Rev Anti Infect Ther 16:187–196. doi:10.1080/14787210.2018.1438263 29415569

[22] Fraser AJ, McMahon FE, Atack JM. 2024. Microbial primer: phase variation - survival and adaptability by generation of a diverse population. Microbiology (Reading) 170:001492. doi:10.1099/mic.0.001492 39222353 PMC11475388

[23] Rappuoli R, Covacci A. 2003. Reverse vaccinology and genomics. Science 302:602. doi:10.1126/science.1092329 14576423

[24] Nabel GJ. 2013. Designing tomorrow’s vaccines. N Engl J Med 368:551–560. doi:10.1056/NEJMra1204186 23388006 PMC3612922

[25] Rappuoli R, Bottomley MJ, D’Oro U, Finco O, De Gregorio E. 2016. Reverse vaccinology 2.0: human immunology instructs vaccine antigen design. J Exp Med 213:469–481. doi:10.1084/jem.20151960 27022144 PMC4821650

[26] Ahearn CP, Kirkham C, Chaves LD, Kong Y, Pettigrew MM, Murphy TF. 2019. Discovery and contribution of nontypeable haemophilus influenzae NTHI1441 to human respiratory epithelial cell invasion. Infect Immun 87:e00462-19. doi:10.1128/IAI.00462-19 31427451 PMC6803334

[27] Johnston JW, Coussens NP, Allen S, Houtman JCD, Turner KH, Zaleski A, Ramaswamy S, Gibson BW, Apicella MA. 2008. Characterization of the N-acetyl-5-neuraminic acid-binding site of the extracytoplasmic solute receptor (SiaP) of nontypeable Haemophilus influenzae strain 2019. J Biol Chem 283:855–865. doi:10.1074/jbc.M706603200 17947229

[28] Murphy TF. 2015. Vaccines for nontypeable haemophilus influenzae: the future is now. Clin Vaccine Immunol 22:459–466. doi:10.1128/CVI.00089-15 25787137 PMC4412935

[29] Pettigrew MM, Ahearn CP, Gent JF, Kong Y, Gallo MC, Munro JB, D’Mello A, Sethi S, Tettelin H, Murphy TF. 2018. Haemophilus influenzae genome evolution during persistence in the human airways in chronic obstructive pulmonary disease. Proc Natl Acad Sci U S A 115:E3256–E3265. doi:10.1073/pnas.1719654115 29555745 PMC5889651

[30] Kovacs-Simon A, Titball RW, Michell SL. 2011. Lipoproteins of bacterial pathogens. Infect Immun 79:548–561. doi:10.1128/IAI.00682-10 20974828 PMC3028857

[31] Narita S-I, Matsuyama S-I, Tokuda H. 2004. Lipoprotein trafficking in Escherichia coli. Arch Microbiol 182:1–6. doi:10.1007/s00203-004-0682-4 15221203

[32] Parsons LM, Lin F, Orban J. 2006. Peptidoglycan recognition by Pal, an outer membrane lipoprotein. Biochemistry 45:2122–2128. doi:10.1021/bi052227i 16475801

[33] Munson RS, Harrison A, Gillaspy A, Ray WC, Carson M, Armbruster D, Gipson J, Gipson M, Johnson L, Lewis L, Dyer DW, Bakaletz LO. 2004. Partial analysis of the genomes of two nontypeable Haemophilus influenzae otitis media isolates. Infect Immun 72:3002–3010. doi:10.1128/IAI.72.5.3002-3010.2004 15102813 PMC387840

[34] Watts SC, Holt KE. 2019. Hicap: in silico serotyping of the haemophilus influenzae capsule locus. J Clin Microbiol 57:e00190-19. doi:10.1128/JCM.00190-19 30944197 PMC6535587

[35] Herriott RM, Meyer EM, Vogt M. 1970. Defined nongrowth media for stage II development of competence in Haemophilus influenzae. J Bacteriol 101:517–524. doi:10.1128/jb.101.2.517-524.1970 5308771 PMC284936

[36] Studier FW. 2005. Protein production by auto-induction in high density shaking cultures. Protein Expr Purif 41:207–234. doi:10.1016/j.pep.2005.01.016 15915565

