# Supplementary material for: Characterization of a conserved outer-membrane protein in non-typeable Haemophilus influenzae with an unidentified impact on phenotype
Source: Microbiol Spectr. 2026 Mar 16;14(4):e02801-25. doi: 10.1128/spectrum.02801-25 (PMC13055389; doi:10.1128/spectrum.02801-25)
Supplement: Supplemental figures and table — Figures S1 to S8 and Table S1. [file spectrum.02801-25-s0001.pdf]

Supplementary Data

Supplementary Table 1 | Primer Sequences	
Primer	Sequence
NTHI1101-flank-F	5′- GGA CGA ACT CTT TTT AGG AAC GC -3′
NTHI1101-flank-R	5′- GCC ACT TTG TTG TTT CAA AAA TTG G -3′
NTHI1101-comp-F	5′- TAT AAT GTT GAT TCA ACC TCT AAT CAT CAT -3′
NTHI1101-outisde-F	5′- GTG TTT TTA TAT CTA ATA TTT AAA GGA AAG AG -3′
NTHI1101-XL-F	5′- ATT GGA TGT GAC GGG CTG GCT CG -3′
NTHI1101-XL-R	5′- CTC GTG ATA CTG ATC CTG CAT TAG GTC A -3′
NTHI1101-OE-F	5′-AGT CAG CAT ATG TGT TCA GAA GAG CAA GTA CAA CGT G -3′
NTHI1101-OE-R	5′- AGT CAG GGA TCC TTA GCC CCC ACT GCT ACG ATG AGT -3′
KanUPout-R	5′- AGA CGT TTC CCG TTG AAT ATG GCT CAT -3′
p601.1-SpecFOUT	5′- CCG TAT GAT TTT AAC TAT GGA CAC GG -3′
T7F	5′- TAA TAC GAC TCA CTA TAG GG -3′
RT_16S-F	5′- ACG GAG GGT GCG AGC GTT AAT C -3′
RT_16S-R	5′- CTG CCT TCG CCT TCG GTA TTC CT -3′
RT_1100_R	5′- TTG CTC CAA CCA CTG CCA TT -3′
RT_1101_F	5′- ACA CCA ACA GGG CAA GTT GT -3′
RT_1101_R	5′- GTA TTT TTA TCT GCC TCA CAA AGC T -3′
RT_1102_F	5′- CAT CAT GAA GCA CGC AAA CTT G -3′
RT_1102_R	5′- TCG GTT GAG CAT TTT GAC GC -3′
RT_1103_F	5′- ATC GCT GAT TTA GCG GTG GG -3′

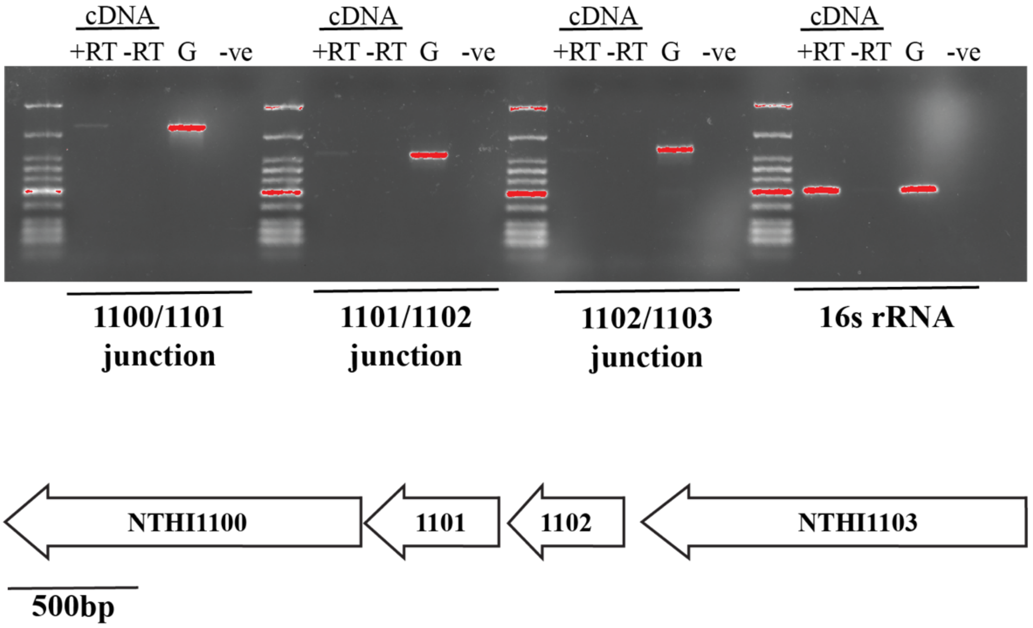

Supplementary Figure 1 | RT-PCR using cDNA made from WT NTHi strain 86-028NP. RNA was prepared using Trizol (Invitrogen) according to the manufacturer's instructions, from mid-log NTHi grown in sBHI broth. cDNA was prepared using Protoscript II reverse transcriptase (NEB) according to manufacturer's instructions. cDNA was diluted 1in10 in water, and 1ul used in a PCR using GoTaq DNA polymerase (Promega) according to manufacturer's instructions and the appropriate primer pair (Supplementary Table 1) as follows: 1100/1101 junction - RT_1100_R+ RT_1101_F; 1101/1102 junction - RT_1101_R+ RT_1102_F; 1102/1103 junction - RT_1102_R+ RT_1103_F. 16s rRNA primers were used as a control to demonstrate cDNA synthesis had occurred, and no genomic DNA was present in the RNA (no signal in -RT control cDNA which had Reverse Transcriptase omitted from cDNA synthesis reaction). Primer sequences are listed in Supplementary Table 1. +RT - reverse transcription using RNA with Protocscript II added; -RT - reverse transcription using RNA without Protocscript II added; G - genomic DNA template as positive control; -ve - no template control for PCR. The arrangement of the four genes in this operon, located on the complementary strand, is illustrated in the schematic below the agarose gel.

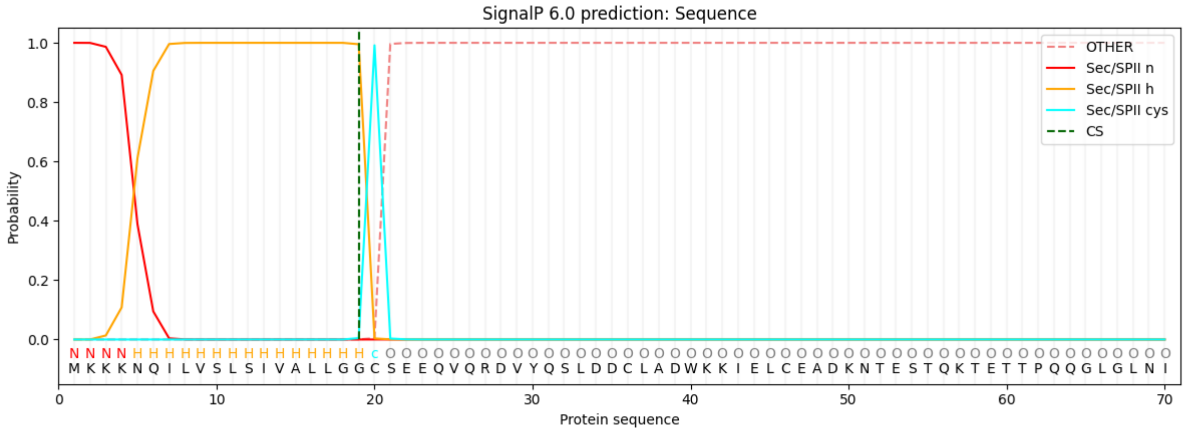

Supplementary Figure 2 | SignalP prediction of NTHI1101 gene. Bioinformatic analysis using SignalP version 6.0 for *NTHI1101* shows Sec/SPII n (red), indicating the likelihood of a signal peptide predicted within the N-terminal region; Sec/SPII h (orange), reflecting the likelihood of a hydrophobic signal peptide; Sec/SPII cysteine (cyan), indicating the probability of a signal peptide containing cysteine; and CS (dashed green), indicating the presence of a cleavage site (source: https://services.healthtech.dtu.dk/services/SignalP-6.0/).

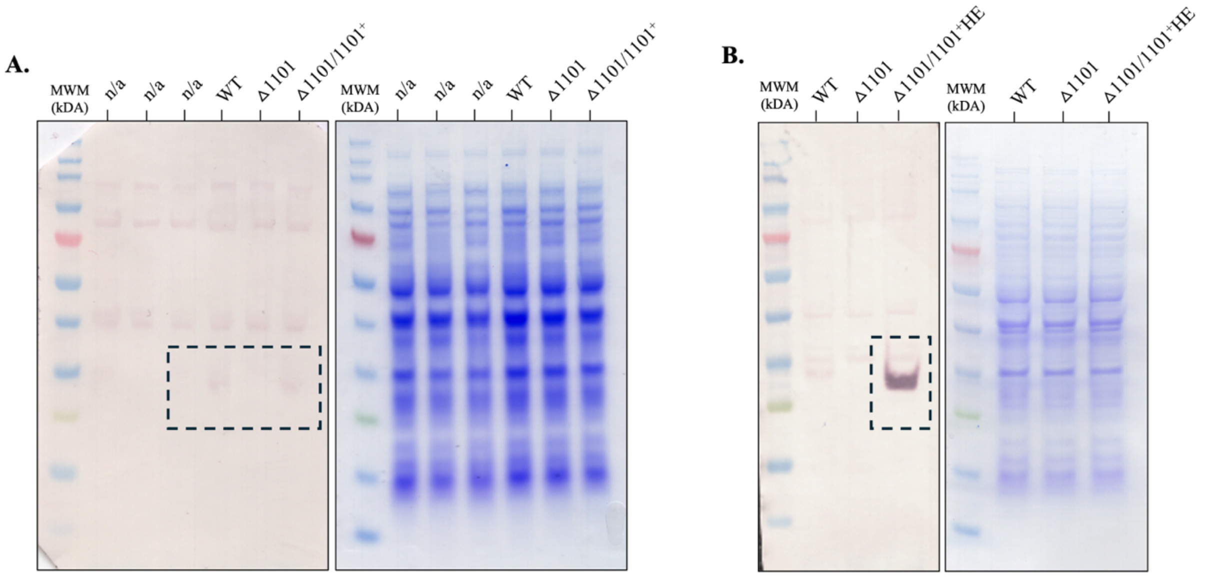

Supplementary Figure 3 | Full images Figure 1 NTHI1101 knockout and complementation strains. A. Western blot (left) and coomassie (right) analysis using whole-cell lysates of WT, △1101 and △1101/1101^{+} strains, adjacent to non-applicable (n/a) samples. The presence of NTHI1101 is detected in the WT and △1101/1101^{+}strain and lack of expression is observed in the △1101 strain. **B.** Western blot (left) and coomassie (right) analysis using whole-cell lysates of WT, △1101 and △1101/1101^{+}HE. The presence of NTHI1101 is detected in the WT and △1101/1101^{+} and a hyper-expression seen in the △1101/1101^{+}HE strain. The Western blots are probed with anti-NTHI1101 antisera at a dilution of 1:500, and secondary antibody at a concentration of 1:10,000.

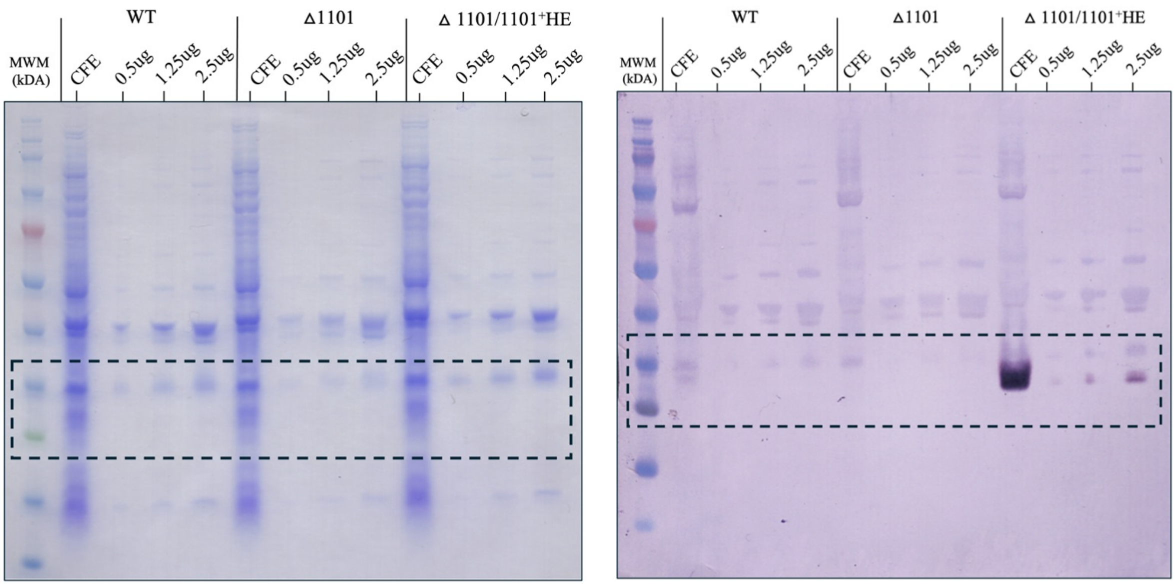

Supplementary Figure 4 | Full image Figure 2 enriched outer-membrane fractions. Coomassie (top) and Western blot (below) analysis using cell free extracts (CFE) and outer-membrane protein (OMP) enriched samples of WT, Δ1101 and Δ1101/1101^{+}HE strains. The Western blot is probed with anti-NTHI1101 antisera at a dilution of 1:500, and secondary antibody at a concentration of 1:10,000. OMP samples were loaded with increasing protein concentrations of 0.5ug, 1.25ug and 2.5ug. The presence of NTHI1101 is detected in the CFE of the WT, as indicated by the black arrow, but is not detectable in the OMP samples. There is complete lack of NTHI1101 in the Δ1101 strain. Hyper-expression of NTHI1101 is observed in the CFE of the Δ1101/1101^{+}HE strain and NTHI1101 is further detected in all OMP samples of the Δ1101/1101^{+}HE strain.

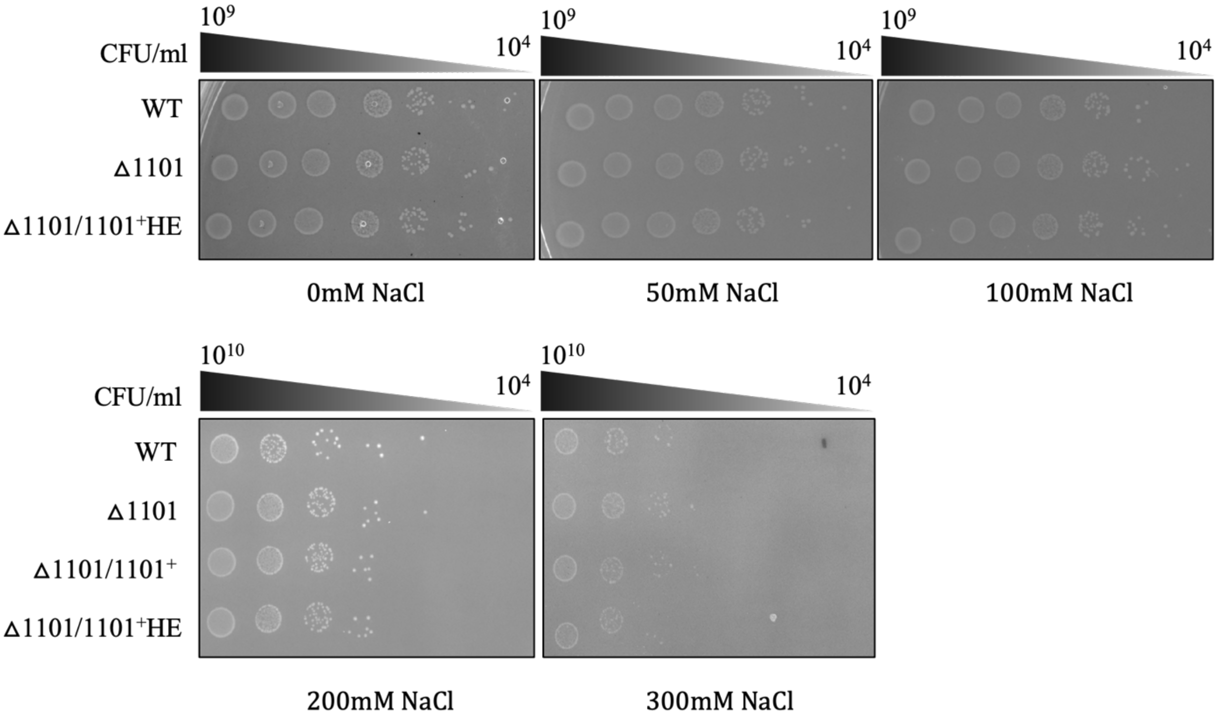

Supplementary Figure 5 | Hyperosmotic assay. Spot viability assay of WT, Δ1101, Δ1101/1101^{+} and Δ1101/1101^{+}HE strains to assess survival in response to hyperosmotic environment. Strains were serially diluted (10^{10} to 10^{4}) and 2ul dot plated onto sBHI agar (top left), sBHI agar supplemented with 50mM NaCl (top middle) or 100mM NaCl (top right) or 200mM NaCl (bottom left) or 300mM NaCl (bottom right). Images were captured with BioRad ChemiDoc imaging System.

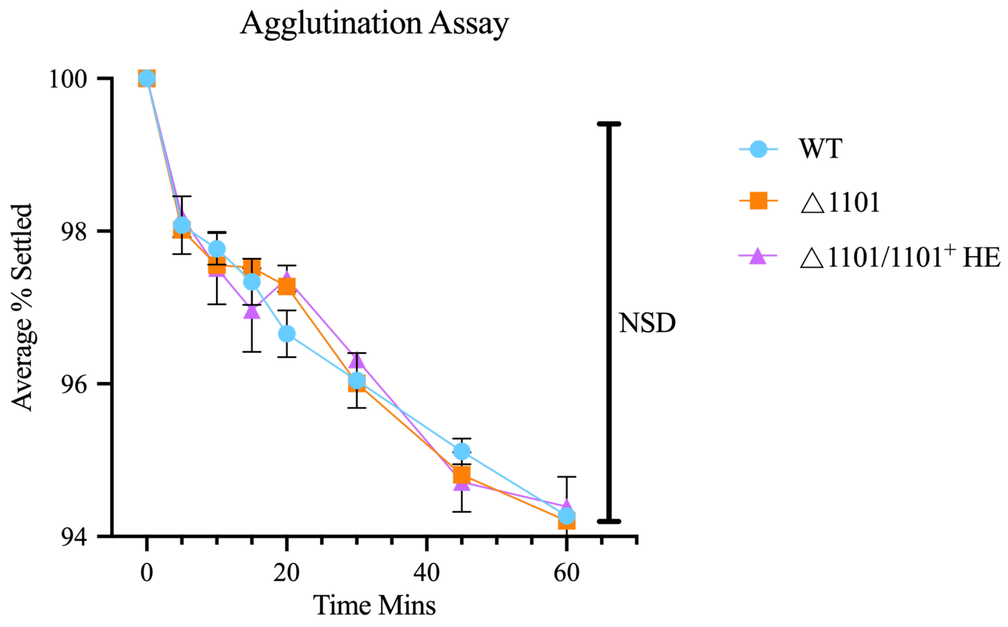

Supplementary Figure 6 | Agglutination assay. The rate of cellular agglutination of WT, ▵1101 and ▵1101/1101^{+}HE strains was examined by monitoring the OD_{600} of static cultures over 60 minutes. Statistical analysis was performed using way of one-way ANOVA. Error bars represent the standard deviation from mean values. NSD indicates no statistical difference between any of the strains.

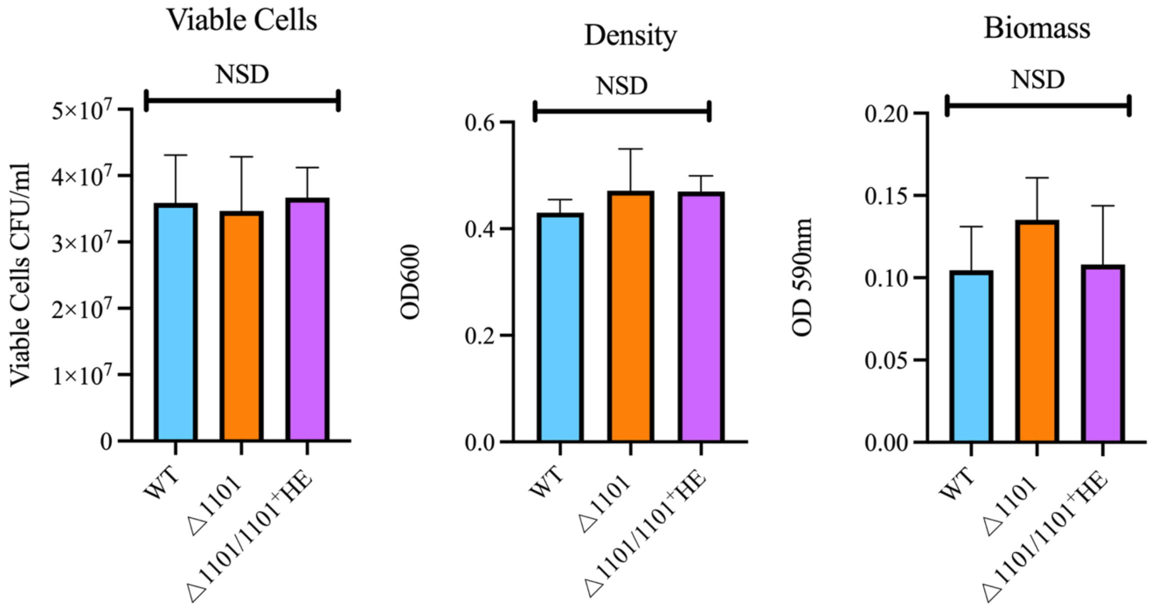

Supplementary Figure 7 | Biofilm assay. The biofilm formation capacity of WT, △1101 and △1101/1101^{+}HE was assessed through measures of viable cells (left), density (middle) and biomass (right) over a 48hr period. Statistical analysis was conducted using an unpaired Students t.test. Error bars represent the standard deviation from mean values. NSD indicates no statistical difference between any of the strains.

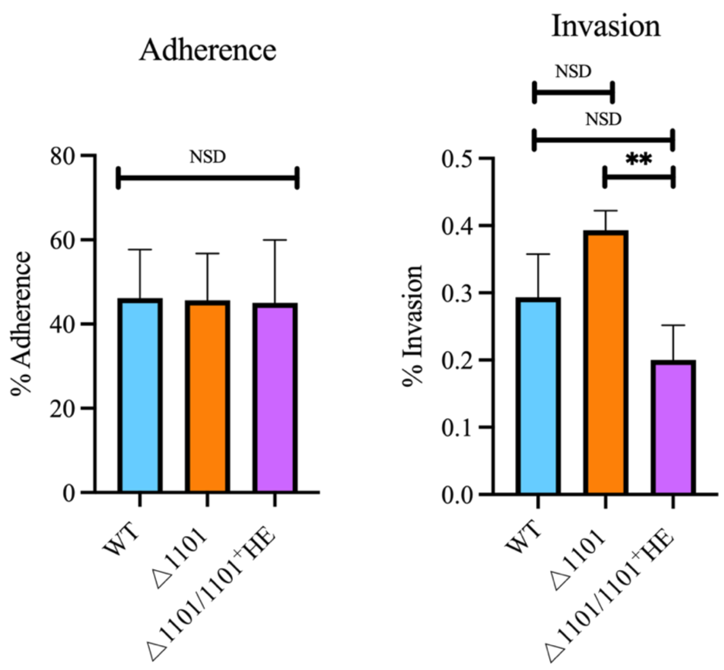

Supplementary Figure 8 | Adherence and invasion assay. The adherence and invasion capacity of WT, △1101 and △1101/1101^{+}HE was assessed using human A459 lung cells. Statistical analysis was conducted using an unpaired Students t.test. Error bars represent the standard deviation from mean values. *P* values were considered significant at <0.05 (*), <0.01 (**), or <0.001 (***). NSD indicates no statistical difference between any of the strains.
